# Identification and expression analyses of *B3* genes reveal lineage-specific evolution and potential roles of *REM* genes in pepper

**DOI:** 10.1186/s12870-024-04897-w

**Published:** 2024-03-19

**Authors:** Young-Soo Park, Hye Jeong Cho, Seungill Kim

**Affiliations:** Department of Environmental Horticulture, University of Seoul, Seoul, 02504 Republic of Korea

**Keywords:** B3, Transcription factors, Solanaceae, Re-annotation, REM, Pepper, Abiotic stress

## Abstract

**Background:**

The *B3* gene family, one of the largest plant-specific transcription factors, plays important roles in plant growth, seed development, and hormones. However, the *B3* gene family, especially the REM subfamily, has not been systematically and functionally studied.

**Results:**

In this study, we performed genome-wide re-annotation of *B3* genes in five Solanaceae plants, *Arabidopsis thaliana*, and *Oryza sativa*, and finally predicted 1,039 *B3* genes, including 231 (22.2%) newly annotated genes. We found a striking abundance of *REM* genes in pepper species (*Capsicum annuum*, *Capsicum baccatum*, and *Capsicum chinense*). Comparative motif analysis revealed that REM and other subfamilies (ABI3/VP1, ARF, RAV, and HSI) consist of different amino acids. We verified that the large number of *REM* genes in pepper were included in the specific subgroup (G8) through the phylogenetic analysis. Chromosome location and evolutionary analyses suggested that the G8 subgroup genes evolved mainly via a pepper-specific recent tandem duplication on chromosomes 1 and 3 after speciation between pepper and other Solanaceae. RNA-seq analyses suggested the potential functions of *REM* genes under salt, heat, cold, and mannitol stress conditions in pepper (*C. annuum*).

**Conclusions:**

Our study provides evolutionary and functional insights into the *REM* gene family in pepper.

**Supplementary Information:**

The online version contains supplementary material available at 10.1186/s12870-024-04897-w.

## Background

The *B3* genes are a superfamily of plant-specific transcription factors. *B3* genes have been characterized as having one or more B3 domains consisting of approximately 110 amino acids, two α-helices, and seven β-sheets [1]. This domain was named because it was first discovered in the third basic domain of the maize *Viviparous-1* (*Vp1*) gene [2]. Based on domain architectures and motifs, *B3* genes are classified into five major subfamilies: ABI3/VP1 [3], ARF (Auxin Response Factor) [4], RAV (Related to ABI3/VP1) [5], REM (Reproductive Meristem) [6], and HSI (High-level expression of sugar-inducible gene) [7, 8]. ABI3/VP1, ARF, RAV, and HSI have been reported to play important roles such as seed development, auxin signaling pathway, flowering time, and maturation [9]. Recently, *ABI3* in Arabidopsis has been reported to control several downstream genes to resist dehydration stress [10]. *ARF* gene is known to be associated with resistance to *Bradyrhizobium* infection [11]. In addition, it was known that ARFs have potential roles in adaptation by regulating soluble sugar content, maintaining chlorophyll content, and promoting root development under salt and drought stresses [12]. RAVs play an important role in plant disease resistance such as cassava bacterial blight [13]. It is known that MED13, which are subunits of the CDK8 module, depend on *HSI* gene to suppress the seed maturation [14]. Although it was recently reported that downregulated *REM34* and *REM35* lead to early arrest of gametophytic development in both male and female Arabidopsis [15], REM still has been recognized as one of the subfamilies and not yet studied in major crops. Recent genomic studies for the *B3* gene family have revealed that a significant presence of *REM* genes within the B3 subfamilies, but the primary focus of these studies has been to investigate the structural chracteristics and phylogenetic relationships within the REM subfamily for classification [16, 17].

The Solanaceae family, which belongs to the asteroid phylogeny of eudicots, includes economically important major crops such as tomato (*Solanum lycopersicum*), pepper (*C. annuum*), and potato (*Solanum tuberosum*). In addition, advances in sequencing technologies have enabled the construction of high-quality genome resources for these species, which are well deposited in public databases [18–21]. Using these resources, the genomic structure and molecular functions of the B3 subfamilies in Solanaceae were also analyzed. Identification of the *B3* gene family including all subfamilies in tobacco has been conducted for exon-intron arrangements, motif conservation, and tissue-specific expression [22]. In pepper and potato genomes, genome-wide studies of ARF family for structural, phylogenetic, and expression profile analyses were performed [23, 24]. Specifically, the function of *ARF* in tomato genome was known to be a defense response through the regulation of the auxin pathway [25]. It was also known that *CaRAV1* has a role as a transcriptional activator that induces resistance to bacterial infection in pepper [26]. However, the evolutionary process and potential roles of *REM* genes in Solanaceae remain unclear.

In this study, we conducted a re-annotation of the *B3* genes in seven plants: *A. thaliana*, *O. sativa*, and five Solanaceae species. The 1,039 *B3* genes were identified, including 231 (22.2%) genes that were omitted in the previous annotation. We found that *REM* genes were mostly abundant in pepper genomes. Through comparative and evolutionary analyses, we classified *B3* genes in the seven genomes into 12 subgroups (G1-12) and identified that a large number of *REM* genes were clustered in the pepper-specific subgroup, G8. The microsynteny, chromosome location, and duplication analyses suggested that the pepper *REM* genes in G8 were recently expanded by lineage-specific tandem gene duplications after the divergence between pepper and other Solanaceae. In addition, expression analyses suggested that pepper *REM* genes are associated with functions under abiotic stresses such as cold, heat, mannitol, and salt. Our results with updated *B3* gene annotations will serve as a fundamental resource for genomic, functional, and breeding research, especially in pepper.

## Results and discussion

### **Annotation update and genomic characteristics of* B3* genes**

**Fig. 1:**
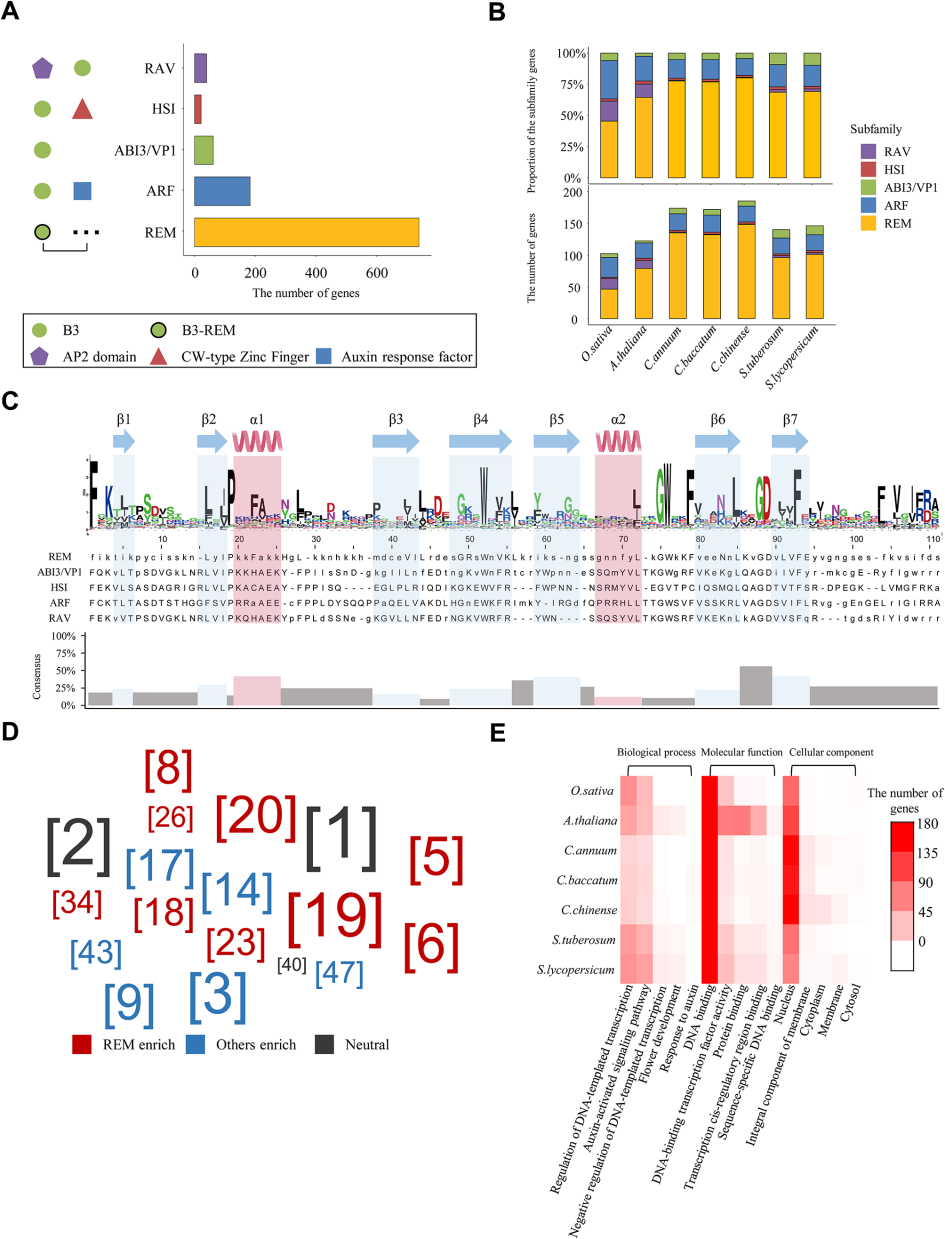
Genomic features of the *B3* genes in seven plant genomes. (**A**) The number of B3 subfamilies and their domain architectures in seven plant genomes. Different symbols located on the left represent the corresponding domains. The color of the bars indicates subfamilies. The dots next to B3-REM express one or more of the B3 domains. (**B**) The ratio and number of subfamilies per species. The bar colors present each subfamily. (**C**) Multiple sequence alignments and secondary structures of B3 domain sequences in seven plant genomes. The secondary structure positions are shown above the amino acid logo. The height of the logos within the stack shows the relative frequency at each position. In the sequence alignment, it is capitalized if more than half of the genes have a particular amino acid at that position, otherwise, it is lowercase. Gaps are marked with dashes. The calculated sequence conservation is presented as a bar diagram. (**D**) Enriched motifs in the B3 domain of REM and other subfamilies. The numbers in brackets manifest the motif sequences shown in Table S4. The size of the numbers shows the frequency of motifs from genes. Colors display the result of the enrichment test for REM and other families (*p* < 0.0001). (**E**) The Gene Ontology (GO) term for updated *B3* genes. The GO categories are listed below the heatmap

We performed re-annotation of the *B3* genes for seven species, including *A. thaliana*, *O. sativa*, and five species of Solanaceae. A total of 1,039 *B3* genes were annotated containing 231 (22.2%) newly identified genes (Table 1). Specifically, we found 75.3% of newly identified *B3* genes in three pepper genomes (*Capsicum* species), ranging from 53 to 67 in individual species. We investigated the domain architectures for *B3* genes and classified them into five known subfamilies: RAV, HSI, ABI3/VP1, ARF, and REM. Specifically, *ABI3/VP1* and *REM* genes had only B3 domain(s), whereas *RAV*, *HSI*, and *ARF* genes had additional AP2, CW-type zinc finger, and auxin response factor domains, respectively, with B3 domain(s) (Fig. 1A). When we examined the ratio and number of *B3* genes from the five subfamilies, *REM* genes were mostly abundant compared to those of other families, especially in three pepper species (Fig. 1B). Specifically, *REM* genes accounted for 78.2% of the newly identified genes in pepper genomes (Table S3). These results indicate that updated *B3* genes from the re-annotation process provide accurate gene repertories of *B3* genes, especially for *REM* genes in pepper genomes.

**Table 1:** The number of re-annotated *B3* genes in seven species

Species	Previously annotated genes	Newly annotated genes	Total
*O. sativa*	96	5	101
*A. thaliana*	116	5	121
*C. annuum*	120	54	174
*C. baccatum*	119	53	172
*C. chinense*	118	67	185
*S. tuberosum*	125	15	140
*S. lycopersicum*	114	32	146
Total	808	231	1,039

To explore the sequence differences of the B3 domain among the five subfamilies, we examined the amino acid sequence of the B3 domain using the updated *B3* genes (Fig. 1C). The B3 domain consisted of approximately 110 amino acids, including two α-helices and seven β-sheets. The position of the first deduced α-helix from the B3 domain was located between the second and third β-sheets, otherwise, the second deduced α-helix was located between the fifth and sixth β-sheets. Given the secondary structure, we subdivided sections of the B3 domain and found that most of the sections were not conserved. Because *REM* genes were particularly variable, we separated *B3* genes in the five subfamilies into two types (REM and other families) and observed high conservation among genes in other families (Fig. S1). This indicates that the *REM* genes have contributed to increased sequence diversity among the B3 domains. We also examined the motif structure of the B3 domain and verified representative motifs abundant in REM or other subfamilies. Motifs #3, #9, #14, and #17 were enriched in other families, whereas motifs #5, #6, #19, and #20 were abundant in REM (Fig. 1D). This implies that REM and other families contain distinct B3 domains and thus may have different functions.

The Gene Ontology (GO) analysis was performed to understand the potential functions of *B3* genes in seven species (Fig. 1E). Many *B3* genes were predicted to be associated with a binding in molecular function and nucleus function in the cellular component. Because transcription factors are generally known to control the transcription of specific genes [27], the *B3* genes were also predicted to play a role as a transcription factor. Taken together, our data generated from the updated *B3* genes provide accurate subfamily repertories, genomic structures, and potential function of *B3* genes in Solanaceae species with *A. thaliana* and *O. sativa*.

### **Motif compositions of REM and other subfamilies of*B3*genes**

**Fig. 2:**
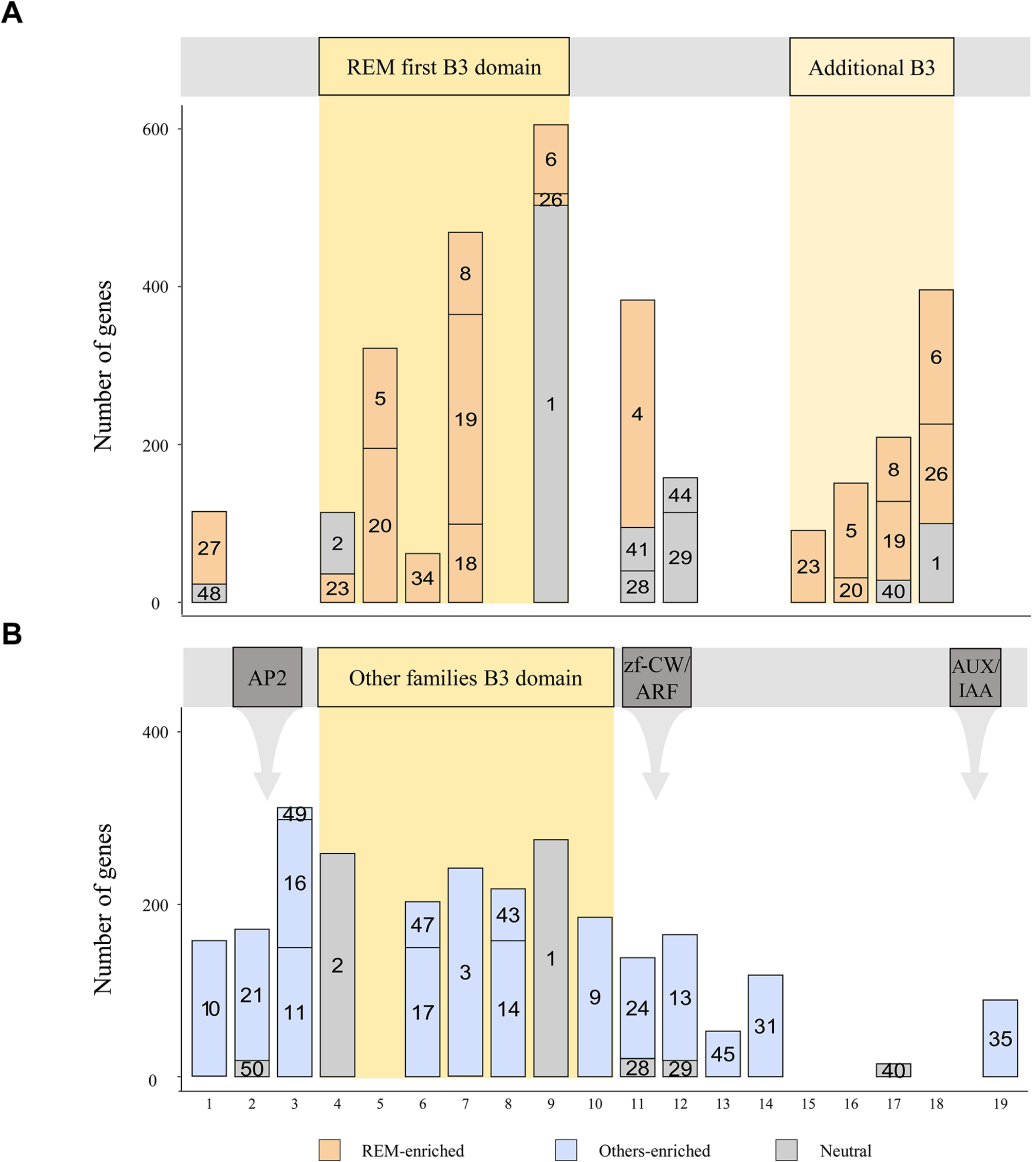
Motif compositions of *B3* genes in seven species. (**A**) The motif compositions of the *REM* genes. If one or more B3 domains are present, only the first B3 is depicted in the REM first B3 domain box, and the remaining B3 domains are illustrated in the additional B3 box. (**B**) The motif compositions of the non-*REM* genes. The integrated domains are shown in the gray box. (**A**-**B**) The number in the bar indicates the motif sequence in Table S4. The result of the enrichment test is displayed as REM-enriched, Others-enriched, or neutral in different colored boxes (*p* < 0.0001)

To compare the motif composition of REM and other families, we analyzed the motif structure and verified 50 conserved motifs in the updated *B3* genes. A total of 36 motifs were mapped at 19 positions, except for 14 motifs that were observed at multiple locations as repetitive motif sequences (Fig. 2). Specifically, two regions of B3 domains were presented in the *REM* genes, at positions 4^th^ to 9^th^ and 15^th^ to 18^th^, respectively (Fig. 2A). However, we found that *B3* genes from other families have a B3 domain located at positions 4^th^ to 10^th^ (Fig. 2B). We also analyzed the non-B3 domain regions and found significant differences between REM and other families mainly due to additional domains in RAV, HSI, and ARF families. These results illustrate distinct amino-acid sequence repertories between REM and other subfamilies. When we examined motif positions in REM or other subfamilies, enriched motifs of *REM* genes were mainly observed in B3 domain regions, whereas the majority of specifically abundant motifs in other subfamilies were positioned in the flanking region as well as within the B3 domain (Fig. 2). These represent that the *REM* genes consisted of different sequences compared to the *B3* genes in other families, and thus may have undergone an independent evolutionary process. Among the B3 domains in the *REM* genes, we found that the first and remaining B3 domain regions have similar motif configurations (Fig. 2A). This suggests that *REM* genes may evolve by gaining repetitive domains.

### **Copy number expansion of specific*REM*genes in pepper**

**Fig. 3:**
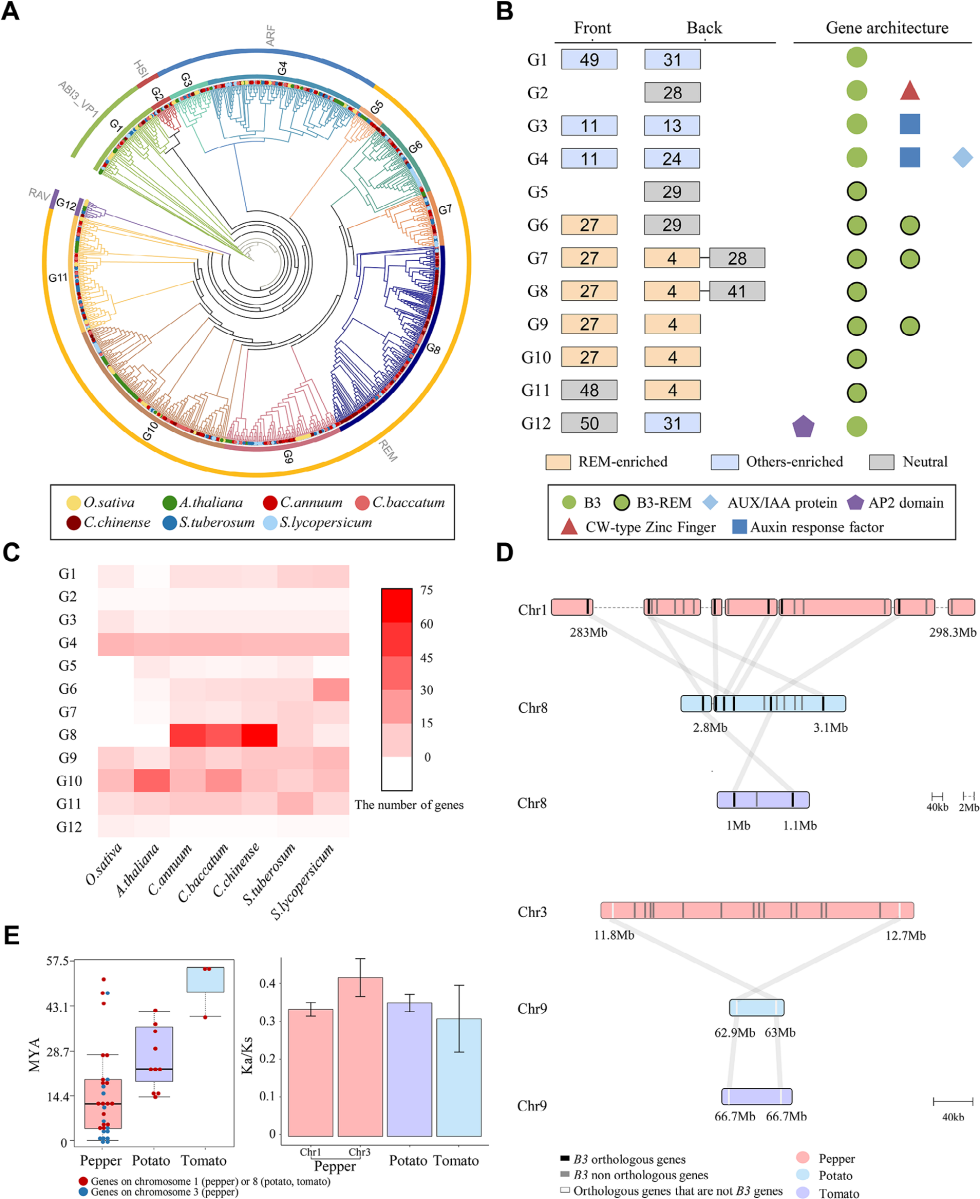
Lineage-specific copy number expansion of *REM* genes in pepper. (**A**) The phylogenetic relationship of the *B3* genes among seven species. The outer and middle rings represent subfamilies and subgroups, respectively. Colored dots at the end of the branch indicate species. (**B**) The most abundant motifs near the B3 domain are shown. Motifs are portrayed as numbers in the box. The boxes are colored to show the type of enriched result of the motifs (*p* < 0.0001). (**C**) The number of subgroups indicated by species on the heatmap. (**D**) Microsynteny relationship of G8 genes in pepper (*C. annuum*), potato, and tomato. The line implies an orthologous relationship between genes. Black and gray blocks mark the chromosome locations of orthologous and non-orthologous *B3* genes. White blocks mean orthologous genes of non-*B3* genes. (**E**) Duplication history of G8 genes in pepper, potato, and tomato. The Ks values of duplication pairs of genes are shown in a dot plot. The Ka/Ks ratio is displayed as a bar graph

To elucidate the evolutionary relationship of *B3* genes, we constructed a phylogenetic tree with 231 updated *B3* genes in the seven plant genomes. Based on the motif compositions of genes and tree branches, we classified 1,010 genes into 12 subgroups (G1-12) (Fig. 3A). Specifically, *REM* genes were constructed into a large lineage that was grouped into G5-11. The motifs in the front and back of the B3 domain were examined to identify the characteristics of each subgroup (Fig. 3B). Specifically, we found that motif #11 was observed within the ARF family, but motifs #13 and #24 were unique to G3 and G4, respectively. This indicates that these motifs contributed to the generation of the genomic diversity of *ARF* genes among those subgroups. Conversely, we observed that dominant motifs among the REM subgroups were conserved overall and shared with motifs #27 and/or #4. However, motif #4 of G7 and G8 had the characteristic of being accompanied by motifs #28 and #41, respectively. The G9 and G10 also showed different characteristics for each subgroup, such as a difference in the number of B3 domains. Based on these conserved structures, this result implies that the duplication of *REM* genes occurred rapidly, resulting in the conservation of genomic sequences of genes in REM subgroups. We then examined the copy number of *B3* genes in each subgroup by species and verified that the overall number of genes in each subgroup of Solanaceae was similar, except for the number of genes in G8 (Fig. 3C). The largest number of genes were congregated in G8, followed by G4. In the G8 subgroup, a large number of *REM* genes (90.64%) were clustered in three pepper genomes, whereas genes in potato and tomato were rarely observed. This suggests that the specific *REM* genes in pepper species have been expanded by lineage-specific evolution, resulting in the conservation of a large pool of *REM* genes in pepper species.

Next, we conducted the chromosome location of the genes for pepper (*C. annuum*), potato, and tomato (Fig. S2). We found that most of the genes were unevenly distributed across 12 chromosomes, suggesting different repertories of *B3* genes among Solanaceae. In particular, the tandem array of genes in G8, located on chromosomes 1 and 3 of the pepper genome, was observed. To compare the genomic regions of the G8 genes on chromosomes 1 and 3 in the pepper genome with the corresponding regions in the Solanaceae genomes, we performed synteny analyses for chromosomes 1 and 3 of pepper and chromosomes 8 and 9 of potato and tomato, respectively (Fig. 3D). Of the 17 and 14 pepper *B3* genes of G8 on chromosomes 1 and 3, respectively, we found only 12.5% and none of the orthologous genes in their corresponding regions in potato and tomato. Because we observed only a few orthologous relationships between the pepper *B3* genes in G8 and the other two species, we assumed that the copy number expansion of the pepper *B3* genes in G8 had recently occurred mainly after speciation. To verify this, synonymous substitution rate (Ks) values were calculated between duplication pairs of G8 in pepper, potato, and tomato, respectively, to estimate the emergence time of genes from G8 in the three species (Fig. 3E). The average MYA of pepper, potato, and tomato were about 14.9 (0.21 Ks), 30.8 (0.43 Ks), and 50.1 (0.7 Ks), respectively. Because 80% of the G8 genes in pepper were smaller than 21.6 MYA (0.3 Ks), we constructed dendrogram based on duplication time for the pepper G8 genes that are located on the chromosomes 1 and 3 to identify how tandem duplications were occurred (Fig. S3). Considering that the divergence point between *Capsicum* and *Solanum* species was around 0.3 Ks [19], our result suggests that the pepper *REM* genes in G8 have been rapidly duplicated and specifically expanded after speciation by tandem duplication, especially in chromosomes 1 and 3. In addition, we also found that pepper G8 genes in chromosome 3 have higher Ka/Ks ratio, suggesting those genes have faster evolutionary rates and thus undergone rapid amino-acid change compared to G8 genes in other species (Fig. 3e). Taken together, our data indicates that pepper-specific copy number expansion of *REM* genes via recent tandem duplication events probably has played a crucial role in the construction of distinct *B3* gene repertoires in pepper compared to other Solanaceae species.

### **Expression and potential roles of pepper*B3*genes under abiotic stress conditions**

**Fig. 4:**
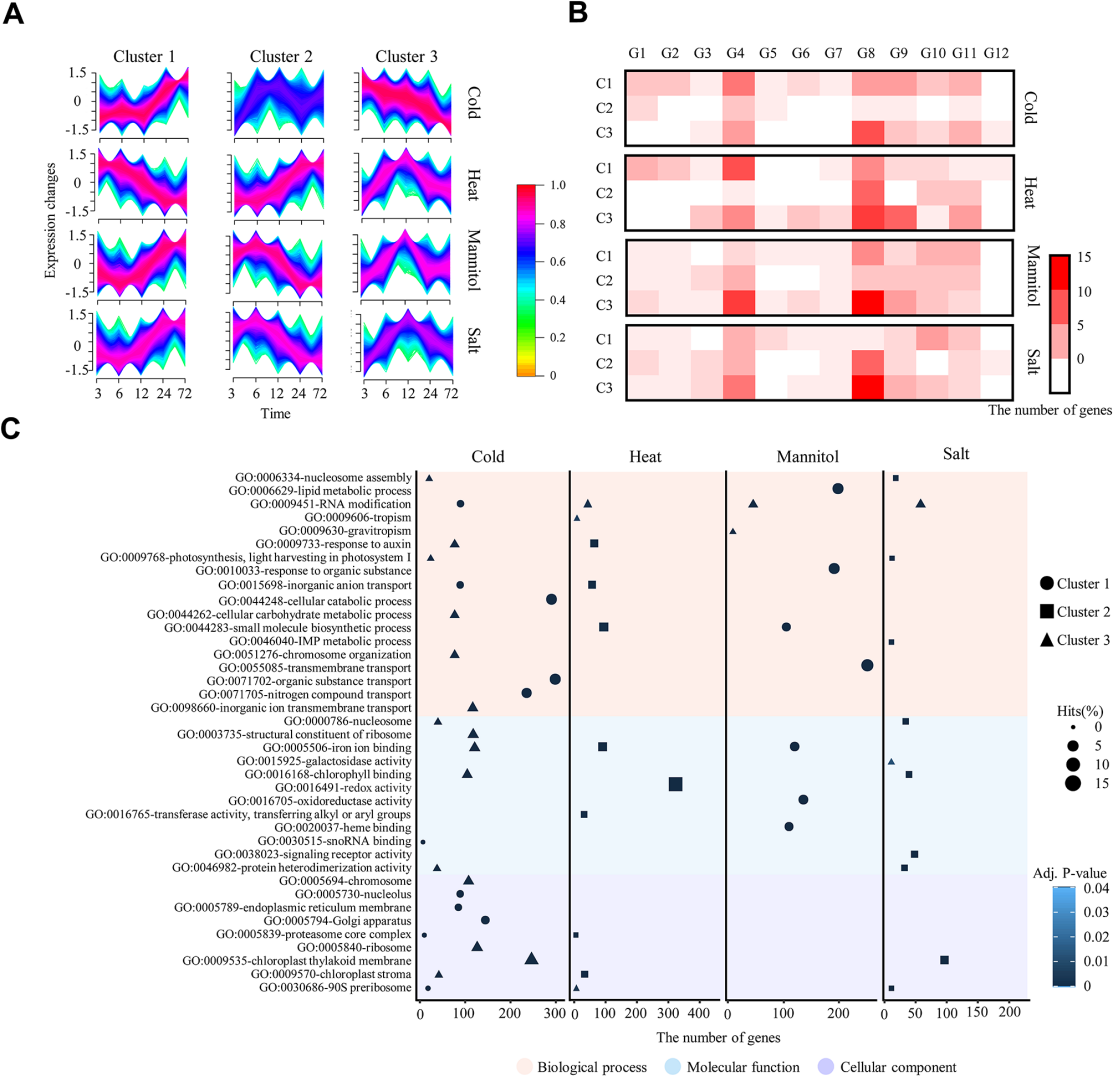
Expression analyses of pepper *B3* genes under abiotic stress. (**A**) Expression patterns of whole DEGs with *B3* genes. (**B**) The number of *B3* genes in expression clusters. The heatmap represents the number of *B3* genes by subgroup. (**C**) Abundant GO descriptions of genes in representative clusters. Dot plots display the top three GO enrichment results for each cluster under abiotic stress. The shape and size of symbols indicate the cluster number and frequency of GO descriptions, respectively, depicted on the right side of the dot plots

To investigate the potential role of the *B3* genes in pepper (*C. annuum*) under abiotic stress conditions, we performed RNA-seq analyses and identified differentially expressed genes (DEGs) under cold, heat, mannitol, and salt stresses. Because previous studies reported the speculation of gene functions by detecting genes that had similar expression patterns with DEGs under certain conditions [28, 29], we also compared the expression of *B3* genes and DEGs and grouped them into three clusters (C1-3) for each stress condition given similar expression patterns (Fig. 4A, Table S5). The expressed *B3* genes were most abundant in mannitol stress with 85 genes, followed by 84 genes, 79 genes, and 69 genes for heat, salt, and cold stress, respectively. This suggests that these *B3* genes, which were similarly expressed with DEGs, may have roles related to each stress. Among the subgroups, we observed many *REM* genes of G8 in clusters, containing 15 genes under cold, 24 genes under heat, 22 genes under mannitol, and 22 genes under salt (Fig. 4B). Specifically, these genes in G8 were abundant in specific clusters. For example, 60% (cold C3), 42% (heat C3), 59% (mannitol C3), and 59% (salt C2) of the *B3* genes were in G8. These suggest that the potential role of pepper-specific expanded *REM* genes is relevant to various abiotic stresses. When we examined the GO terms of whole genes in two clusters by each abiotic stress condition, including abundant *REM* genes in G8, we observed various abiotic stress-related functions, such as response to auxin (GO:0009733) in cold and heat, small molecule biosynthetic process (GO:0044283) in heat and mannitol, and signaling receptor activity (GO:0038023) in salt (Fig. 4C). In addition, many genes in specific clusters, such as cold C1, heat C3, mannitol C3, and salt C3, were associated with an RNA modification (GO:0009451) encoding a pentatricopeptide repeat (PPR) domain. Our results suggest the potential role of pepper *B3*, especially *REM* genes in G8 under abiotic stress conditions via association with a variety of other genes.

### **Co-expression network and functional association of pepper*REM*genes under abiotic stress conditions**

**Fig. 5:**
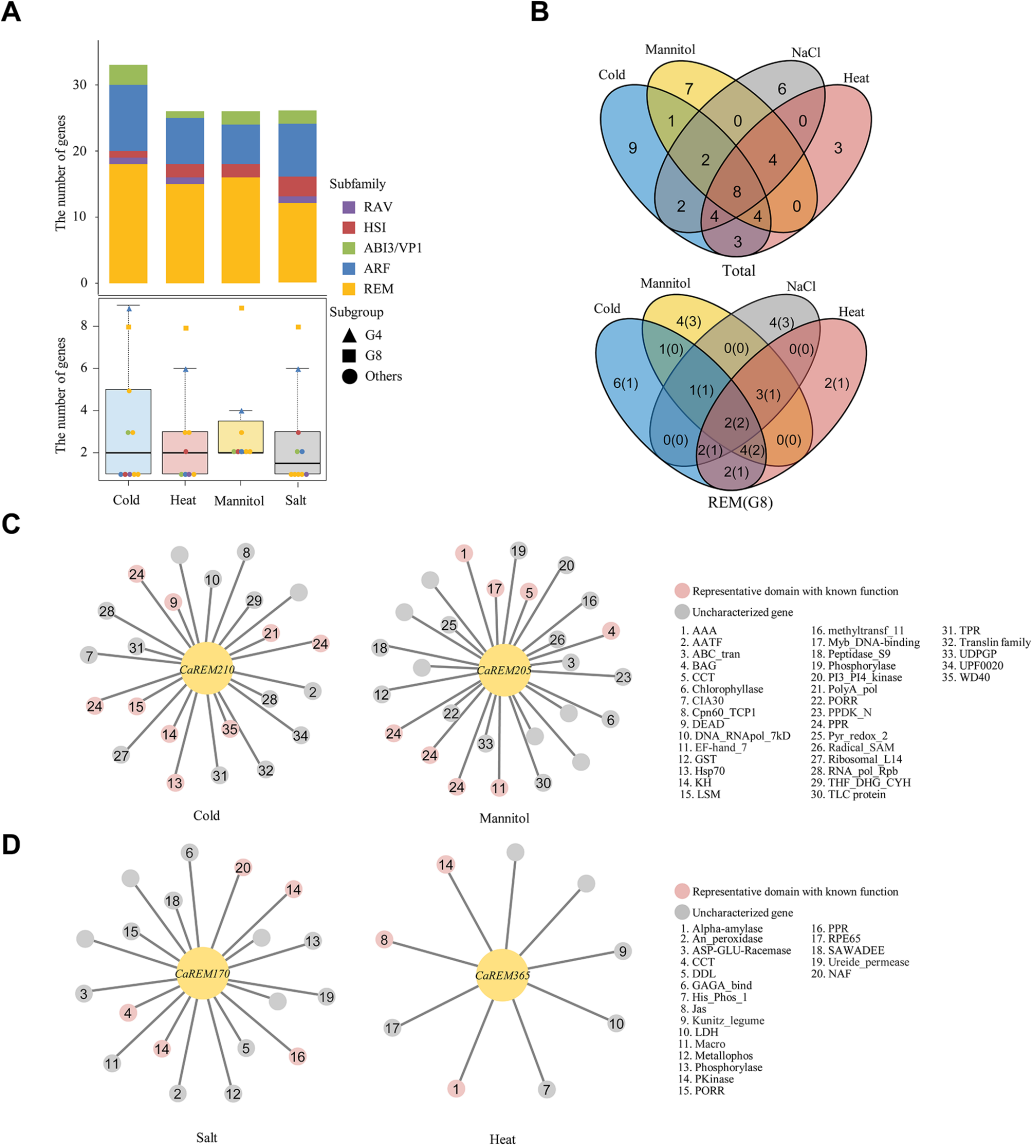
Co-expression network analysis of *REM* genes in pepper under abiotic stress. (**A**) The number of *B3* DEGs under each abiotic stress. Colors and shapes next to the diagram symbolize the subfamilies and subgroups. (**B**) DEGs of the *B3*, *REM*, and G8 genes. (**C**) The networks illustrate associations with G8 genes and other genes under cold and mannitol conditions. (**D**) Co-expression network of stress response-related *REM* genes under salt and heat (**C**-**D**). The number written in the circle provides domain information, which is located on the right side of the network. The empty circle represents an uncharacterized gene

We detected *B3* DEGs in expression clusters and verified an overall similar distribution across four stresses: 33 (cold), 26 (heat), 26 (mannitol), and 26 (salt). Of these, the *REM* DEGs, especially in the G8, were mostly abundant regardless of the stresses (Fig. 5A). Furthermore, we identified 25 stress-specific DEGs, and 16 (8) of them belonged to REM (G8) (Fig. 5B). Co-expression networks of target genes suggest their potential roles given the repertories of linked genes in the expression network [30]. We constructed co-expression networks of stress-specific *REM* DEGs with other DEGs in the same expression clusters to understand the specific roles of pepper *REM* genes under abiotic stress conditions. Our analyses revealed that pepper *REM* genes in G8 could be involved in cold and mannitol stress-induced functions of various genes (Fig. 5C, Table S6). In particular, we detected that *CaREM210* and *CaREM205* were co-expressed with three *PPR*s and a variety of genes under cold and mannitol conditions, respectively. Previous studies have reported the functions of *PPR*s under cold and mannitol conditions. The repressed expression of *TCD10* (*LOC_Os10g28600*) and *OsV4* (*LOC_Os04g39970*) genes in rice caused abnormal chloroplast development at low temperatures [31, 32]. *SOAR1* (*At5g11310*) in Arabidopsis was the positive response to particularly cold and osmotic stress through the regulation of ABA signaling [33]. In addition to the *PPR* genes, *OsRH42* (*Os08g0159900*), which encodes a DEAD-box helicase in rice, is important for pre-mRNA splicing under cold stress [34]. The *Rboh* gene (*LOC107862088*, *LOC107875997*), encoding several domains such as EF-hand, NADPH oxidase, and FAD-binding in pepper, was known to be activated by the binding of Ca cations to the EF-hand, causing the accumulation of malondialdehyde in response to mannitol stress [35]. Our data suggest that pepper *REM* genes in G8 could be involved in the regulation of cold and mannitol stress-related traits with *PPR* and other various genes via examination of co-expressed genes with pepper *REM* genes in G8 under those stress conditions.

Furthermore, we observed diverse genes that co-expressed with pepper *REM* genes in G8 and G9 under salt and heat stress (Fig. 5D, Table S6). In salt stress, we also found genes involved in the regulation of ABA [33, 36], the regulation of ROS production [37], and the early events of the signal transduction pathway [38] such as CCT, PPR, and protein kinase domain, as described in previous studies. For example, overexpression of *AtCOL4* (*At5g24930*), which encodes the CCT domain in Arabidopsis, is known to regulate ABA synthesis and stress-related genes under salt stress [36]. These results suggest that the pepper *REM* genes in G8 may play a role in the salt stress condition through co-expression with other salt stress-related genes. In heat stress, *CaREM365* in G9 was co-expressed with nine genes having various domains such as protein kinase, α-amylase, and lactate/malate dehydrogenase, suggesting the putative role of the pepper *REM* gene in G9 with these genes. Taken together, our data comprehensively suggest the underlying roles of pepper *REM* genes, especially those belonging to the pepper-specific expanded G8 via association with a variety of genes involved in abiotic stress responses based on the investigation of DEGs and co-expression networks.

## Conclusions

Construction of gene annotations without omission of genes that existed in genome assemblies is a crucial step for gene family studies [39–43]. In this study, we performed an annotation update of the *B3* genes and identified 231 new gene models in five Solanaceae, *A. thaliana*, and *O. sativa* genomes. Notably, newly annotated genes in pepper accounted for 78.2% of novel *REM* genes. The motif analyses of the updated *B3* genes showed the different amino acid composition of B3 domains between *REM* and other *B3* genes, indicating that they may have undergone independent evolutionary processes. Based on the phylogenetic relationship, we divided the *B3* genes into 12 subgroups and found a lineage-specific burst of pepper *REM* genes in G8. These pepper *REM* genes in G8 comprised 50.6% of the 174 newly annotated pepper *B3* genes, indicating the significantly improved *B3* gene annotation of the pepper genome through the identification of pepper-enriched *REM* genes in this study. These pepper *REM* genes in G8 were mainly clustered on chromosomes 1 and 3. These pepper *REM* genes were overall non-syntenic with *REM* genes in corresponding regions of the tomato and potato genomes. These also indicated pepper-specific evolution of those *REM* genes via recent tandem duplication after speciation between pepper and other Solanaceae species.

Because our analyses suggested that pepper *REM* genes, especially in G8, could be pepper-specific genes and thus related to pepper-specific traits, we focused on RNA-seq analyses mainly for *REM* genes in pepper (*C. annuum*) under cold, heat, mannitol, and salt stress conditions. We found that the DEGs in expression clusters, including abundant *REM* genes, were overall involved in RNA modification in response to abiotic stresses. Furthermore, the co-expression regulatory networks of stress-specific *REM* genes suggested that specific *REM* genes in G8, in particular, were co-expressed with previously known functional genes such as several PPRs under cold and mannitol stresses [28–30]. Consequently, our results with the updated *B3* genes provide insights into the genomic structural features, evolutionary history, and potential roles of pepper *B3* genes, including pepper-specific evolved *REM* genes.

## Materials and methods

### **Re-annotation of the*B3*gene family in seven plant genomes**

Data for genome sequences of *A. thaliana* [44], *O. sativa* [45], *C. annuum* [19], *C. baccatum* [20], *C. chinense* [20], *S. tuberosum* [21], and *S. lycopersicum* [18] were downloaded for re-annotation of *B3* genes. Re-annotation of *B3* genes was conducted using TGFam-Finder v1.20 [46], which tool was developed to focus on identification of missing genes in annotations based on protein mapping, transcriptome data, and ab initio prediction. As described in the previous study [46] with parameters ‘EXTENSION_LENGTH’ = 200,000, ‘MAX_INTRON_‌LENGTH’=200,000, ‘HMM_CUTOFF’=1e-4 (Table S1). The TSV file was created from InterProScan 5 (-f tsv -appl Pfam) [47] using “TSV_FOR_DOMAIN_IDENTIFICATION”, and the target ID was set to PF02362 (B3) by the PFAM (http://pfam.xfam.org/) and Hidden Markov Model (HMM) databases. We used several Pfam IDs such as PF06507 (Auxin response factor domain), PF02309 (AUX/IAA family), PF00847 (AP2 domain), and PF07496 (CW-type zinc finger domain) to separate the B3 subfamilies. Finally, we assigned new names to the updated *B3* genes instead of using the published annotation names (Table S2).

### Conserved amino acids in the B3 domain

The acid sequence of the B3 domains was extracted for the seven plant genomes. To align the B3 domain sequences, we utilized MAFFT v7.470 (--reorder --maxiterate 1000) [47], and then TrimAL v1.4 [48] with the gt 0.2 parameter to trim the alignment. We visualized the amino acid sequence composition of all B3 domains using WebLogo v2.8.22 [49]. To elucidate the consensus sequence of the B3 domain according to the subfamily, the EMBOSS Cons program [50] was used with the plurality 0.1 option. Multiple alignments were performed using Jpred (default parameters) [51] to predict the secondary protein structure of the B3 domain. Sequence conservation score was calculated for 19 regions of B3 domain divided given the α-helix and β-sheet structures of B3 domains. Average consensus scores for each division were calculated by MACSIMS from Jalview programs [52].

### Statistical test for enrichment

To identify which motif is specifically enriched in genes belonging to REM or others (ABI3/VP1, ARF, RAV, and HSI), we used Fisher’s test and Chi-square test using an in-house Perl script executed with the Statistics::R module R. *P*-values were computed by the Monte Carlo test (B = 10,000).

### **Gene Ontology term of*B3*genes**

Functional annotation of *B3* genes was conducted using OmicsBox v2.2.4 [53]. To align B3 protein sequences to the NCBI database for non-redundant protein database (nr v5), BLASTP was used with a 1e-3 e-value cut-off. Blast2GO mapping and annotation were performed using the Blast results that were merged with the InterProScan results [54]. Next, we divided the GO terms of each *B3* gene into three categories (biological process, molecular function, and cellular component). From direct GO, the top five GO terms for each category were displayed.

### **Motif compositions of*B3*genes**

To determine the conserved motifs of the *B3* genes, MEME v5.1.1 (-protein -mod zoops -nmotifs 50 -minw 10 -maxw 50 -objfun se -markov_order 0) [55] was conducted. A total of 50 motifs were matched to protein sequences by MAST v5.1.1 [56]. Conserved motif positions were determined manually based on sequence alignments and motif compositions, except for recurring motifs at various positions.

### **Phylogenetic analysis of*B3*genes**

The 1,039 re-annotated *B3* genes were aligned using MAFFT v7.470 [47]. Ambiguous alignments were removed with TrimAL v1.4 with trim option gt 0.1 [48]. To infer phylogenetic relationships, the maximum likelihood tree was constructed using IQ-TREE v2.0.6 [57] with bootstrap replicates of 1,000 ultrafast parameters. Interactive Tree of Life (iToL) v6 was utilized to visualize the tree. The final tree of *B3* genes was organized into 12 subgroups (G1-12) based on domains and motifs.

### **Chromosome distribution and microsynteny of*B3*genes in pepper, potato, and tomato**

The physical locations of the *B3* genes, excluding unanchored scaffolds, were determined from the GFF files generated by TGFam-Finder v1.20 [46]. We used Mapchart to show the distribution of *B3* genes on the chromosomes [58]. Subgroups of all genes were represented in different colors according to the phylogenetic tree.

To show the orthologous relationships of the G8 genes, microsynteny analysis was performed. All-by-all comparison of BLASTP [59] and GFF files obtained by TGFam-Finder v1.20 [46] results were identified using the Multiple Collinearity Scan toolkit (MCScanX) [60] with parameters such as a match score 50 and a match size 3. We used RIdeogram in R packages to represent the genomic locations for each gene pair [61].

### Duplication history of G8 genes in pepper, potato, and tomato

Duplicated pairs of *B3* genes were identified using DupGen_Finder [62], and the duplication time of those pairs was calculated. Multiple alignments were conducted with PRANK (-codon) using the coding sequences of each gene pair. We estimated the non-synonymous substitution rates (Ka) and synonymous substitution rates (Ks) of each duplicated *B3* gene pair using KaKs_Calculator 2.0 (-m MYN) [63]. The evolutionary tree was constructed using the Ks value between pepper G8 genes based on median linkage hierarchical clustering of hclust of the R package to determine the order of duplication. To calculate million years ago (MYA), we used the formula T = Ks/2λ. Each of λ was assumed to be 6.96 × 10^−^^9^ [64–66]. We displayed the duplication time with Beeswarm in R packages.

### **Transcriptome analyses of pepper and tomato*B3*genes under abiotic stresses**

We first downloaded RNA sequencing data of pepper under abiotic stresses for cold, heat, mannitol, and salt at different times:3 h, 6 h, 12 h, 24 h, and 72 h from the NCBI Sequence Read Archive (SRP187794; Table S7) [67]. To eliminate low-quality RNA sequencing data, trimming was performed by CLC Assembly Cell (CLC Bio, Aarhus, Denmark) using fastq raw files. Next, we conducted HISAT2 (-dta -x) [68] and StringTie (-e -B -G) [69] to map the *C. annuum* reference genome and calculate the fragment per kilobase transcript per million mapped reads (FPKM) values ​​of the updated *B3* genes. To convert FPKM values to read counts, we used Python scripts (prepDE.py). We examined differentially expressed genes (DEGs) with DESeq2 in R software with |log2FoldChange| >1 and adjusted *p*-value < 0.05 [70]. Clustering analyses were completed on *B3* genes and DEGs from Mfuzz [71] programs in R packages with log2(FPKM + 1) under abiotic stress. Three clusters of each stress were determined according to the k-means algorithm of the Mfuzz package in the R software with K = 3, selected as the predetermined number of clusters. GO annotation was then retrieved with OmicsBox v2.2.4 [53] for clusters containing abundant *REM* genes in G8. In addition, we carried out an enrichment test of GO terms from Fisher’s exact test (false discovery rate corrected *p*-value ≤ 0.01) and showed more specific GO terms with the reduction to the most specific option. Co-expression network analysis based on expression clusters was performed using WGCNA [72] in R packages with optimal β (soft thresholding power) values selected by SFT.R.sq over 0.8 for all stress conditions and minModuleSize of 30. We visualized networks using the Cytoscape v3.9.1 program.

### Electronic supplementary material

Below is the link to the electronic supplementary material.

Supplementary Material 1

Supplementary Material 2

## Data Availability

The datasets provided in this study can be detected online, and the accession numbers are written in the article or additional files.

## Abbreviations

ARF: Auxin Response Factor
RAV: Related to ABI3/VP1
REM: Reproductive Meristem
HSI: High-level expression of sugar-inducible gene
GO: Gene Ontology
DEG: Differentially expressed gene
FPKM: Fragment Per Kilobase of transcript per Million mapped reads
