# Supplementary material for: Identification and expression analyses of B3 genes reveal lineage-specific evolution and potential roles of REM genes in pepper
Source: BMC Plant Biol. 2024 Mar 19;24:201. doi: 10.1186/s12870-024-04897-w (PMC10949715; doi:10.1186/s12870-024-04897-w)
Supplement: Supplementary file 1 — Supplementary Material 1 [file 12870_2024_4897_MOESM1_ESM.pdf]

Supplementary Figure 1

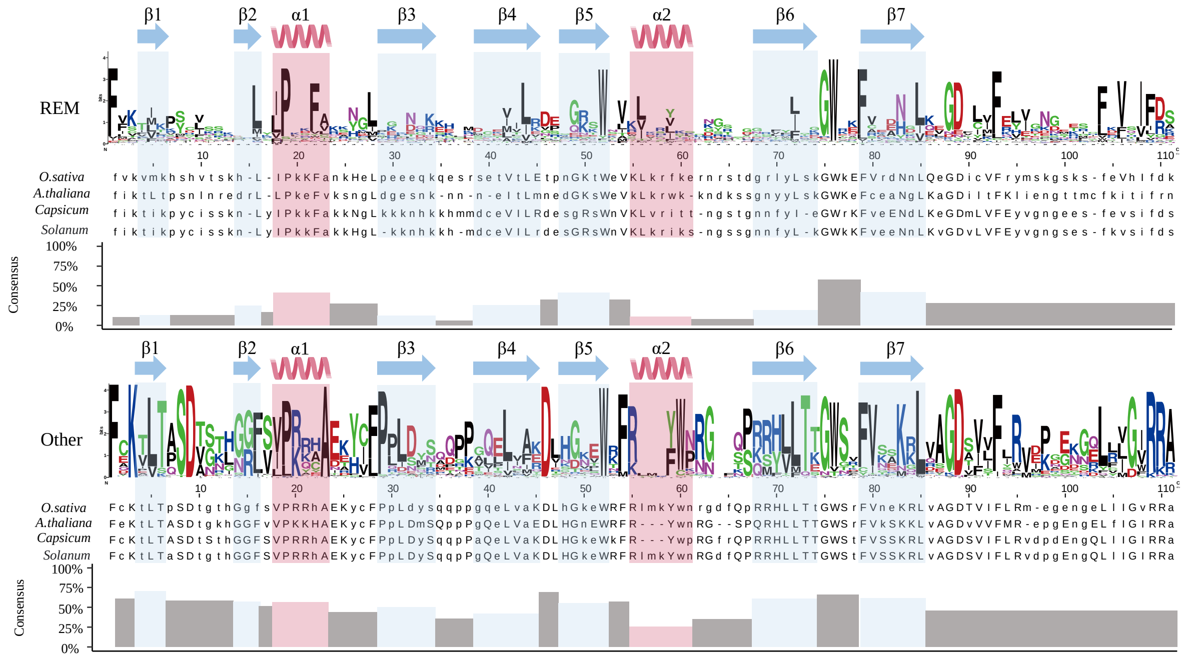

Fig. S1. Comparison of amino acid sequence alignment for REM and other (ABI3/VP1, ARF, RAV, and HSI) families. The α-helices and β-sheets are presented above the amino acid logo. Each logo consists of stacks of symbols indicating the most conserved amino acid for each position. The height of the logos within the stack shows the relative frequency at each position. In the sequence, it is uppercase if more than half the genes have that amino acid, otherwise, it is lowercase. Gaps are marked with dashes. The calculated sequence conservation is presented as a bar diagram based on the secondary structure positions.

Supplementary Figure 2

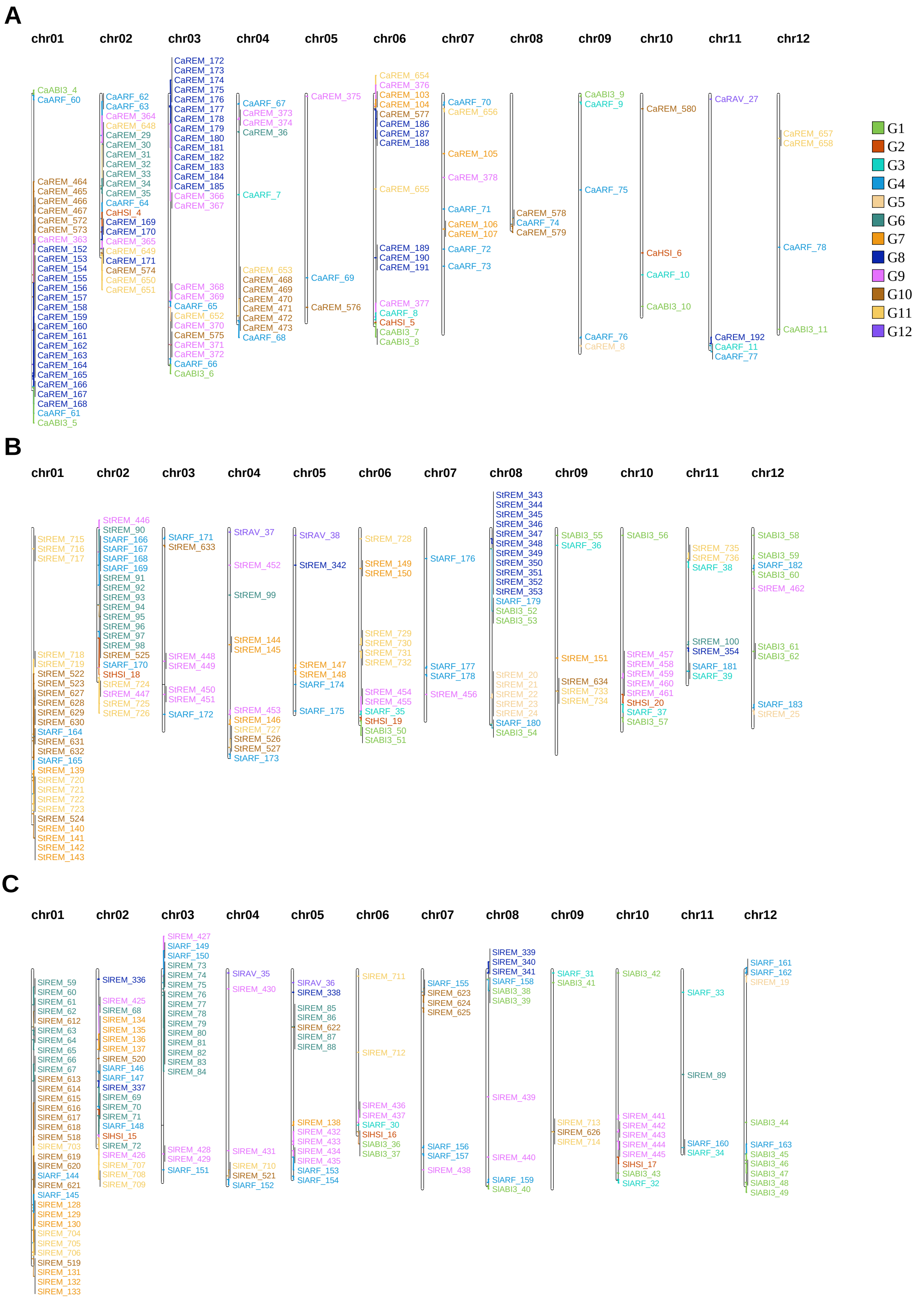

Fig. S2. Chromosomal locations of *B3* genes in pepper (*C. annuum*), potato, and tomato. (A-C) (A), (B), and (C) show the distribution of *B3* genes for pepper, potato, and tomato in this order. The genes are denoted by different colors based on the subgroups. The tandem array genes are connected to the black lines to the left of the gene names.

Supplementary Figure 3

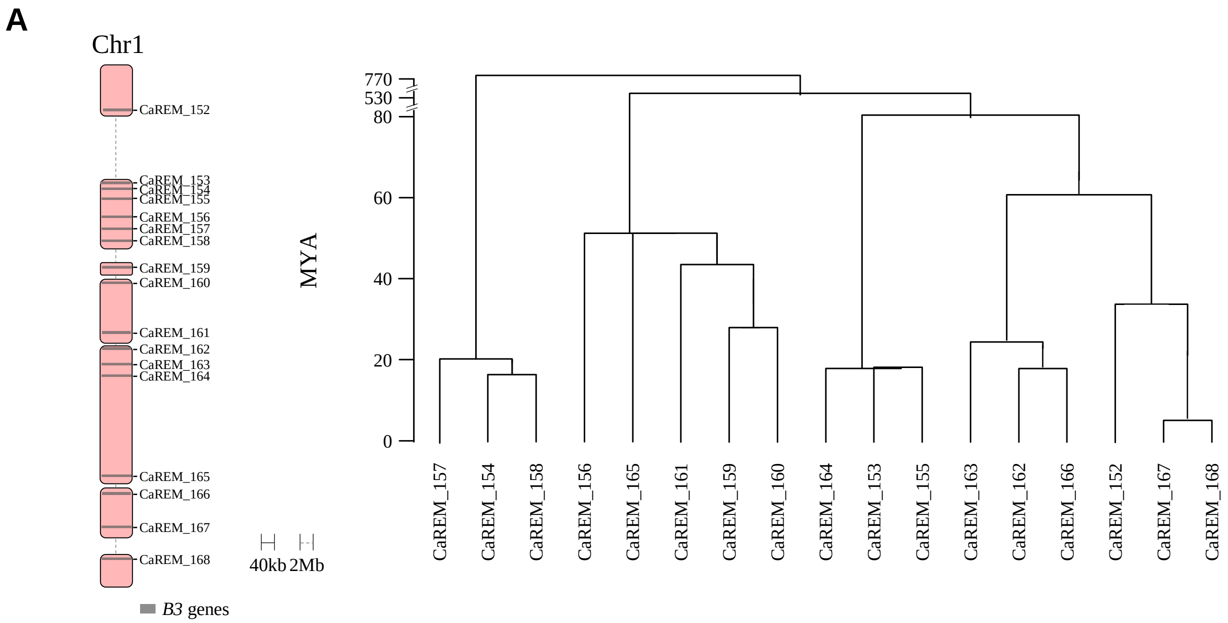

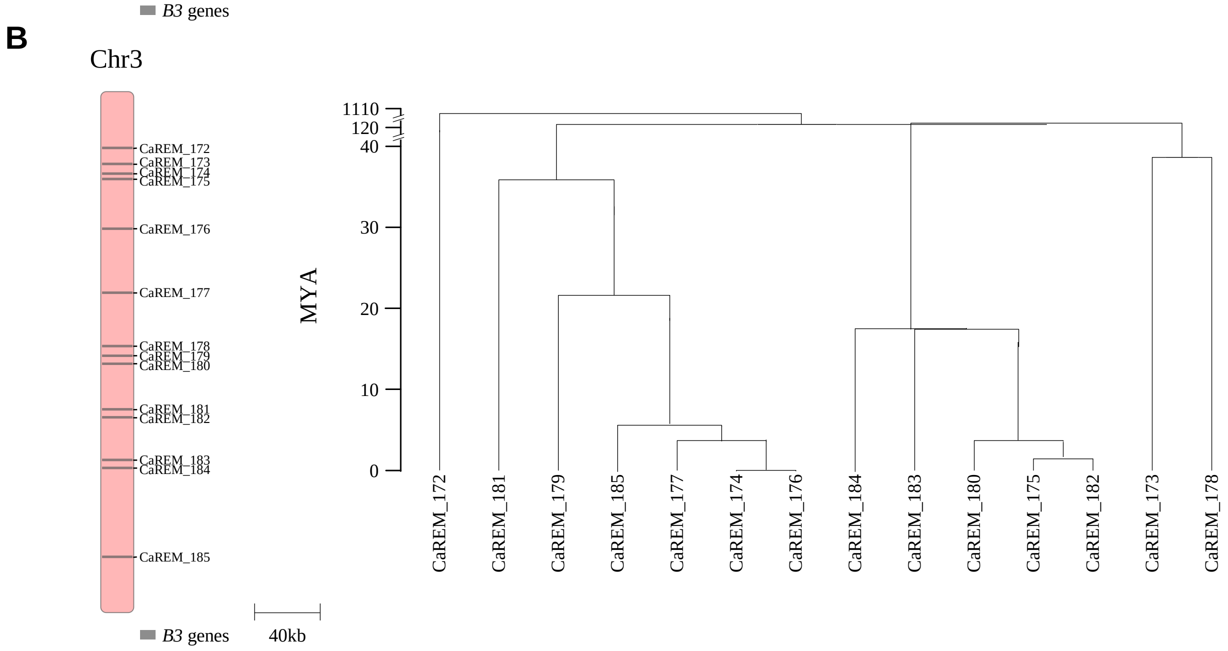

Fig. S3. Predicted the time and order of tandem duplication on chromosomes 1 and 3 for pepper G8 genes via hierarchical clustering. Gray blocks mark the chromosome locations of pepper G8 genes. The duplication history of G8 genes is shown in the dendrogram plot.
